# “Adaptive learning” as a mechanistic candidate for reaching optimal task-set representations flexibly

**DOI:** 10.1186/1471-2202-15-S1-P8

**Published:** 2014-07-21

**Authors:** Salva Ardid, Matthew Balcarras, Thilo Womelsdorf

**Affiliations:** 1 Department of Biology and Centre for Vision Research, York University, Toronto, Ontario, Canada, M3J 1P3

## 

Top-down inputs from prefrontal cortex impact on sensory neurons [1,2], enhancing their selectivity to attended stimuli, while sensory processing of distractors is suppressed [1,3,4]. However, what are the neuro-computational mechanisms that identify the behaviorally relevant information that is worth to bias? Previous studies linked selective attention to learned value [5], suggesting that attentional selection relies on internal mechanisms that track the relevance of sensory information. Consistent with this view, we recently introduced a reinforcement learning (RL) approach for the deployment of selective attention [6]. We tested model-free and model-based versions of RL to identify the mechanisms that most accurately predict behavior: whereas model-based prioritizes attentional selection to task features that are systematically associated with reward, model-free considers all available features. Our results proved that the optimal task-set representation significantly improved predictive power, suggesting that monkeys benefited from model-based mechanisms [6]. Yet, model-based presents two important limitations: i) it is unable to adapt to changes in the association of reward with sensory features that are not included in the learned model, and ii) it excludes the mechanism of learning by which subjects derive the proper task-set representation. The question remains then of how a prioritized task set can be learned to exploit the benefits of employing a model.

With the aim to workaround model-based limitations and shed light on the underlying mechanisms that make model-based benefits possible, we propose here the “adaptive learning” mechanism for flexible task-set representation. The mechanism is dynamically tuned according to the statistics of association between sensory features and reward outcome, flexibly adjusting its regime of operation between model-free and model-based systems. To test the adaptive learning mechanism, we developed it in a RL model and extended our previous analysis of monkey behavior to both, cued [5] and uncued [6], versions of the same attention task. This RL model was able to transition from a naive starting point to an optimal task-set representation, and to flexibly adapt among optimal task-set representations upon changes in reward contingencies. The model achieved so by tracking a separate learning rate α for each stimulus feature in the environment. If the selection of a stimulus feature was systematically correlated with a particular outcome (reward or no-reward), α increased. In contrast, when a feature was unable to consistently predict reward, α decayed. Thus, α changed dynamically, and over a large number of trials any α associated with a non-predictive feature went to zero, effectively eliminating it from the task set, which tuned performance towards that of the model-based system.

The adaptive learning mechanism introduced here represents a step further in our understanding of the origins of selective attention. Notably, our results prove that the adaptive learning is an optimal mechanistic candidate to support arbitrary prioritized model-based formation under generic conditions of covert attentional selection, and regardless of whether attentional selection operates on cued or uncued tasks.

## References

[B1] Ardid S Wang X-J Compte A An integrated microcircuit model of attentional processing in the neocortex J Neurosci 2007 15 32 8486 8495 10.1523/JNEUROSCI.1145-07.2007 17687026 PMC6672949

[B2] Gregoriou GG Gotts SJ Zhou H Desimone R High-frequency, long-range coupling between prefrontal and visual cortex during attention Science 2009 15 5931 1207 1210 10.1126/science.1171402 19478185 PMC2849291

[B3] Reynolds JH Chelazzi L Attentional modulation of visual processing Annu Rev Neurosci 2004 15 611 647 10.1146/annurev.neuro.26.041002.131039 15217345

[B4] Maunsell JH Treue S Feature-based attention in visual cortex Trends Neurosci 2006 15 6 317 322 10.1016/j.tins.2006.04.001 16697058

[B5] Kaping D Vinck M Hutchison RM Everling S Womelsdorf T Specific contributions of ventromedial, anterior cingulate, and lateral prefrontal cortex for attentional selection and stimulus valuation PLoS Biol 2011 15 12 e1001224 10.1371/journal.pbio.1001224 22215982 PMC3246452

[B6] Balcarras M Ardid S Kaping D Everling S Womelsdorf T Learning of expected stimulus values in a selective attentional foraging task by nonhuman primates Soc Neurosci Abstr 2012

